# Reduced 5-hydroxymethylcytosine due to TET2 downregulation is associated with chondrosarcoma progression

**DOI:** 10.1038/s41598-025-25820-9

**Published:** 2025-11-25

**Authors:** Hiroshi Furukawa, Takeshi Iwasaki, Kengo Kawaguchi, Hiroki Sonoda, Kenichi Kohashi, Hidetaka Yamamoto, Yasuharu Nakashima, Yoshinao Oda

**Affiliations:** 1 Department of Anatomic Pathology, Graduate School of Medical Sciences, Kyushu University, 3-1-1 Maidashi, Higashi-ku, Fukuoka, 812-8582 Japan; 2 Department of Pathology, Graduate School of Medicine, Osaka Metropolitan University, Osaka, Japan; 3 Department of Pathology and Oncology, Okayama University, Okayama, Japan; 4 Department of Orthopaedic Surgery, Graduate School of Medical Sciences, Kyushu University, 3-1-1 Maidashi, Higashi-ku, Fukuoka, 812-8582 Japan

**Keywords:** DNA methylation, Chondrosarcoma, 5-hydroxymethylcytosine, Ten-eleventranslocation gene, MAPK signaling pathway, PI3K/AKT/mTOR pathway, Bone cancer, Sarcoma, Bone cancer

## Abstract

Chondrosarcoma (CS) is the second most common malignant bone tumor, known for its poor prognosis, high recurrence rate, and potential for metastasis. Current prognostic predictive methods rely heavily on histological grading, underscoring the need for additional markers. DNA methylation, particularly the role of 5-hydroxymethylcytosine (5hmC), has emerged as a promising prognostic indicator in various malignancies. This study aimed to investigate the relationship between 5hmC expression levels and CS prognosis. Additionally, RNA analysis was performed to identify differentially expressed genes associated with low 5hmC levels. The findings indicate that low 5hmC levels were significantly linked to a worse prognosis and decreased ten-eleven translocation 2 (TET2). Furthermore, the reduction in 5hmC levels was linked to the activation of oncogenic signaling pathways, such as MAPK and PI3K-Akt/mTOR. Immunohistochemical markers such as phospho-MEK (p-MEK), p-ERK, p-Akt, and p-mTOR were higher in the 5hmC low group than in the high group. These findings suggest that 5hmC levels not only reflect the epigenetic state of CS tumors but also hold promise as a valuable prognostic marker for identifying patients at heightened risk of adverse outcomes. Furthermore, TET2 downregulation results in a decrease in 5hmC levels with subsequent activation of the MAPK and PI3K/Akt/mTOR pathways.

**Supplementary Information:**

The online version contains supplementary material available at 10.1038/s41598-025-25820-9.

## Introduction

Chondrosarcoma (CS) is the second most common primary malignant bone tumor after osteosarcoma and is characterized by a poor prognosis, often resulting in local recurrence and distant metastasis. Histopathologically, conventional CS exhibits atypical chondrocytes with nuclear atypia, nuclear pleomorphism, and binucleation, along with a chondroid matrix and permeative patterns. CS is categorized into grades 1 to 3 based on nuclear atypia and cell density, according to the histological criteria outlined in the World Health Organization (WHO) classification of soft tissue tumors^1–3^. Besides histological classification, there are currently no other indicators to assess CS malignancy. Therefore, additional factors that can predict prognosis are needed.

DNA methylation is an essential epigenetic mechanism that regulates gene expression, with DNA demethylation playing a crucial role in promoting gene expression^4^. The DNA demethylation process is orchestrated by two key epigenetic modifiers: DNA methyltransferases (DNMT) and ten-eleven-translocation (TET) enzymes^5^. DNA methylation primarily occurs at cytosine-guanine (CpG) sites, where DNMT attaches a methyl group to cytosine, forming 5-methylcytosine^6^. Subsequently, TET enzymes oxidize 5-methylcytosine to generate 5-hydroxymethylcytosine (5hmC), which can further be converted into 5-formylcytosine and 5-carboxylcytosine^5,7^. The intermediate product of this process, 5hmC, is stably expressed in many tissues and is being investigated as a marker reflecting the state of DNA methylation. It has been reported that 5hmC levels are significantly reduced in various cancers, such as breast cancer and colorectal cancer^8^. However, a previous study investigating variations in 5hmC levels among CS cases found that they were not associated with IDH1/2 mutations^9^. Hence, the prognostic significance of 5hmC itself remains unclear.

This study investigated the relationship between 5hmC levels and the prognosis of conventional CS cases. It also explored the factors contributing to the decrease in 5hmC expression and investigated cancer signaling pathway activation as a consequence.

## Material and methods

### Case selection

A total of 45 CS cases (36 cases of conventional CS and nine cases of dedifferentiated CS) that were diagnosed at the Department of Anatomic Pathology, Graduate School of Medical Sciences, Kyushu University, Fukuoka, Japan, between 1978 and 2020 were investigated. All cases underwent surgical resection and histological examination and were classified into three grades according to the 2020 WHO Classification of Tumors of Soft Tissue and Bone^1^. Conventional CS was diagnosed based on the presence of either apparent nuclear atypia or bone destruction (permeating pattern). Cases with only biopsy specimens, as well as those involving non-conventional types of CS such as secondary CS (Ollier disease and multiple osteochondroma), periosteal CS, clear cell CS, and mesenchymal CS, as well as cases receiving preoperative chemotherapy or radiotherapy, were excluded. We examined a full-face section of the tumor across the longest axis of the tumor. The average number of tumor blocks submitted for diagnosis was 17 (3–50).

### Clinicopathological analysis

**Fig. 1:**
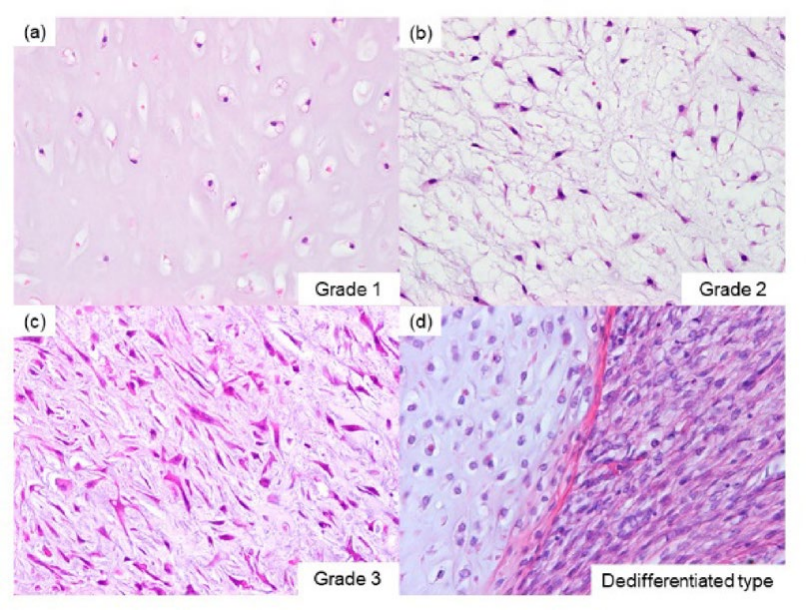
Representative histological figure of chondrosarcoma. Grade 1 with mild nuclear atypia and increased cellularity (**a**), grade 2 with moderate nuclear atypia and cellularity (**b**), grade 3 with high cellularity, marked atypical cells, and pleomorphic appearance (**c**), and dedifferentiated type with a conventional chondrosarcoma component and an abrupt transition to a high-grade non-cartilaginous sarcoma (**d**).

CS was histologically classified based on the current WHO classification, which considers nuclear pleomorphism, cellularity, and mitotic activity. Grade 1 malignancy displays minimal nuclear pleomorphism and high cellularity, grade 2 malignancy exhibits moderate nuclear pleomorphism and cellularity, and grade 3 malignancy shows increased cellularity with prominent atypia and pleomorphic appearance. Dedifferentiated CS is characterized as a high-grade subtype with a conventional CS component transitioning abruptly to a high-grade, non-cartilaginous sarcoma (Fig. 1). Survival data were analyzed for 45 cases of CS (36 cases of conventional CS and nine cases of dedifferentiated CS), with follow-up periods ranging from 0 to 395 months (mean 67, median 35). Clinical outcomes were assessed based on age, gender, tumor location, and history of local recurrence, distant metastasis, and tumor-related death. Tumor locations were categorized as appendicular skeleton (humerus, femur, tibia, fibula, and phalanges) and nonappendicular skeleton (vertebrae, ribs, sternum, clavicle, pelvis, and scapula).

### DNA extraction

Genomic DNA was extracted from fresh-frozen specimens following the manufacturer’s protocols for all procedures, as previously described^10^. The frozen specimens were scraped and stained with hematoxylin and eosin to confirm the presence of malignant components.

### RNA extraction

The total RNA was extracted from fresh-frozen specimens and formalin-fixed paraffin-embedded (FFPE) specimens using the MagMAX™ mirVana Total RNA Isolation Kit and RNAstorm ™ FFPE RNA Extraction Kit (Cell Data Science). For the FFPE specimens, each of the grades 1–3 CS and dedifferentiated CS samples was collected by scraping under visual inspection. Frozen specimens were scraped and stained with hematoxylin and eosin to confirm the presence of malignant components.

### Next-generation sequencing for targeted genes

DNA was extracted from 13 frozen tissue samples, including three grade 1, seven grade 2, one grade 3, and two non-dedifferentiated components of dedifferentiated CS. Next-generation sequencing (NGS) was performed using an amplicon-based approach. Library preparation was performed using an Ion AmpliSeq™ On-Demand Panel for targeted sequencing of 13 genes (Thermo Fisher Scientific, Waltham, MA) and an Ion AmpliSeq™ Library Kit Plus (Thermo Fisher Scientific). Emulsion polymerase chain reaction and enrichment were performed using an Ion OneTouch™ 2 System (Thermo Fisher Scientific). Sequencing was performed on the Ion PGM™System (Thermo Fisher Scientific) with an Ion 318™ Chip Kit v2 BC (Thermo Fisher Scientific). Raw sequence data were processed using Ion Reporter software (Thermo Fisher Scientific; https://ionreporter.thermofisher.com/ir/), with filtering parameters set as follows: coverage ≥ 50; allele frequency ≥ 0.01; variant effect: excluding synonymous variants.

### mRNA expression profiling and bioinformatics analysis

In total, 12 samples were extracted from 4 frozen specimens and 8 FFPE specimens, including two grade 1, three grade 2, and one grade 3 samples, in addition to three dedifferentiated and three non-dedifferentiated components from the same dedifferentiated CS cases. The nuclear grade of the tumor cells was histologically confirmed. RNA quality was assessed using the Agilent 2100 Bioanalyzer (Agilent Technologies), and DV200 values were subsequently analyzed to evaluate RNA integrity. Comprehensive mRNA expression was assessed using the nCounter® Gene Expression Assay (NanoString Technologies Inc.). The extracted mRNA samples were enriched for the target regions of the nCounter® Human PanCancer Pathways™ Panel and analyzed using the nCounter® Analysis System (NanoString Technologies Inc.).

Differentially expressed genes between the high and low 5hmC groups, with |log2 (fold change)|≥ 1 and a *p*-value < 0.05, were identified using the DESeq2 package in R (version 4.3.1; R Foundation for Statistical Computing). Gene Ontology and Kyoto Encyclopedia of Genes and Genomes (KEGG) pathway enrichment analyses were performed and graphically presented using ShinyGO version 0.80 (http://bioinformatics.sdstate.edu/go/)^11^. The significance threshold for enrichment was set at a false discovery rate of ≤ 0.05.

### Immunohistochemistry

For each case, the resection section with the highest nuclear grade was selected, and FFPE specimens were prepared for immunohistochemical (IHC) studies. Primary antibodies used are summarized in Supplementary Table S1. Nuclear staining for 5hmC, TET1, TET2, TET3, and DNMT3B was assessed using the H-score, calculated as follows: 1 × (% of cells with 1 + staining) + 2 × (% of cells with 2 + staining) + 3 × (% of cells with 3 + staining), yielding a continuous scale ranging from 0 to 300. Cutoff values were determined based on mean and median values, with cutoffs set at ≥ 120 for 5hmC and TET2, ≥ 80 for TET1, and ≥ 220 for DNMT3B, classifying them into high and low groups. Succinate dehydrogenase B (SDHB) was considered positive when tumor cells exhibited granular cytoplasmic staining (Supplementary Fig. S1).

Phospho-Akt, phospho-mTOR, phospho-MEK1/2, and phospho-ERK1/2 expression were evaluated based on staining intensity in the nucleus or cytoplasm compared to that in the endothelial cells, as previously reported^12,13^. The proportion of stained cells was scored from 0 to 3, with 0 indicating 0% stained cells, 1 indicating 1%–9%, 2 indicating 10%–49%, and 3 indicating 50%–100%. Scores of 2 and 3 were considered positive. Immunostaining results were compared using a *t*-test, with a *p-*value of < 0.05 considered statistically significant.

### 5hmC quantification

5hmC content in DNA was quantified using an enzyme-linked immunosorbent assay (ELISA)-based MethylFlash Hydroxymethylated DNA Quantification Kit (Epigentek), following the manufacturer’s instructions. Absorbance at 450 nm was measured using a Multiskan FC microplate reader (Thermo Fisher Scientific). The standard curve slope was determined using linear regression, and the percentage of 5hmC in total DNA was calculated using the following formula:

$$5hmC\% = \frac{{{\text{Sample}}\;{\text{OD}} - {\text{Negative}}\;{\text{control }}\;{\text{OD}}}}{{{\text{Slope}} \times {\text{Input}}\;{\text{DNA}}\;{\text{amount}}}} \times 100\%$$

The correlation between 5hmC levels and the 5hmC H-score was evaluated using Spearman’s rank correlation coefficient.

### Survival analysis

Statistical analyses were performed using R version 4.3.1 (R Foundation for Statistical Computing). Survival curves for overall survival (OS) and progression-free survival (PFS) were generated using the Kaplan–Meier method and compared between groups using log-rank analysis. Statistical significance was set at a *p*-value of < 0.05. OS was defined as the time from tumor resection to death, while PFS was defined as the time from tumor resection to the detection of distant metastasis or local recurrence. Survival curves were estimated using the Kaplan–Meier method and compared using the log-rank test. Hazard ratios (HR) and 95% confidence intervals (95% CIs) were calculated using the Cox univariate regression model.

Variables that showed significant differences in the univariate analysis (*p* < 0.05) were included in the multivariate analysis.

### Ethical approval

The study was conducted in accordance with the Declaration of Helsinki and approved by the Institutional Review Board of Kyushu University (approval number 23005-02). Informed consent was obtained from all participants, and patient data confidentiality was guaranteed.

## Results

### Clinicopathological characteristics

Clinical and pathological findings are summarized in Table 1. Among the 45 cases of CS, there were 26 males and 19 females, with a median age of 51 years (interquartile range [IQR]: 38–66). According to the current WHO classification, there were 11 cases of grade 1, 23 cases of grade 2, and two cases of grade 3. The median tumor size was 7 cm, and this value was used as the cutoff to stratify the cases into two groups. Additionally, there were nine cases of dedifferentiated CS. The follow-up period ranged from 1 to 395 months, with a mean of 67 months and a median of 35 months.

**Table 1:** Clinicopathologic characteristics.

Factor	Group	n = 45 (%)
Age	≧50	12 (26.7)
	< 50	33 (73.3)
Sex	M	26 (57.8)
	F	19 (42.2)
Tumor location	Appendicular skeleton*	32 (71.1)
	Nonappendicular skeleton†	13 (28.9)
Tumor size	≧ 7 cm	15 (33.3)
	< 7 cm	20 (44.4)
	uncertain	10 (22.2)
Histological grade	1	11 (24.4)
	2	23 (51.1)
	3	2 (4.4)
	Dedifferentiated type	9 (20.0)
Necrosis	+	25 (55.6)
	-	20 (44.4)

*Appendicular skeleton (humerus, femur, tibia, fibula, and phalanges).

†Nonappendicular skeleton (vertebrae, rib, sternum, clavicle, pelvis and scapula).

### Frequent IDH1 mutations detected on targeted next-generation sequencing

**Fig. 2:**
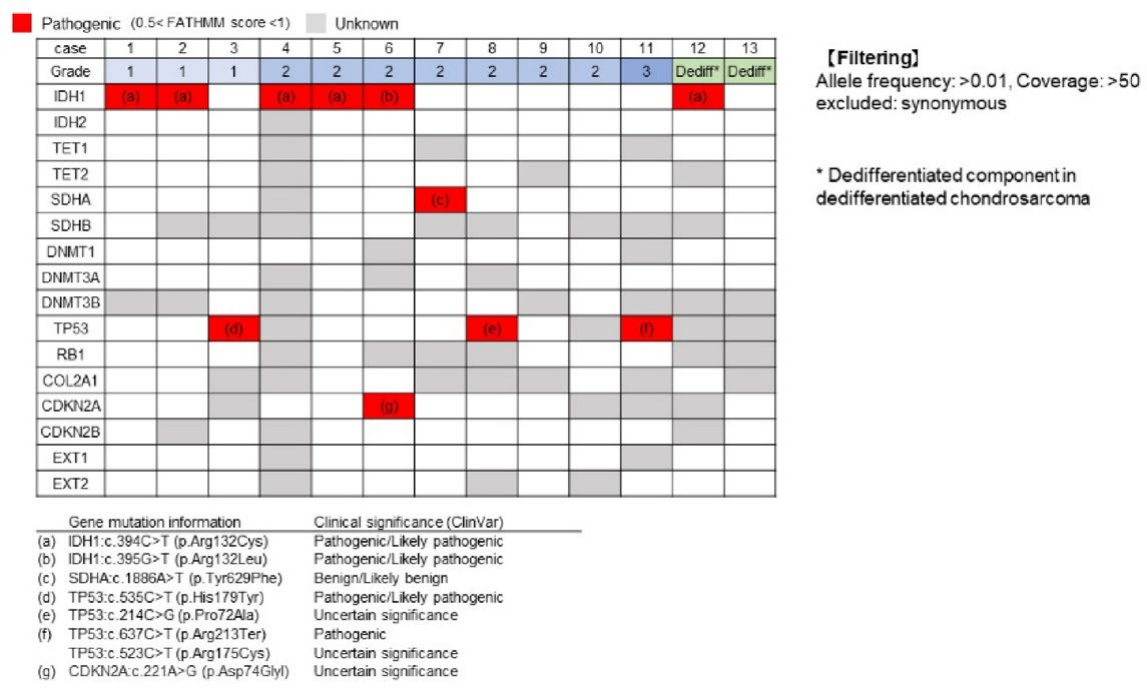
Commutation plot of gene alterations. *IDH1* mutations were identified in six out of 13 cases (46%), while *p53* mutations were identified in three cases. No pathogenic mutations were identified in the *TET*, *DNMT*, *SDHA*, and *SDHB* genes.

Somatic variants are depicted in Fig. 2. Pathogenic mutations in the *IDH1* gene were detected in six out of 13 cases (46%), with c.394C > T in five cases and c.395G > T in one case. These variants impact the Arg132 residue located in the active site of the enzyme crucial for isocitrate binding in the catalytic pocket. No somatic mutations were identified in the *TET*, *DNMT*, *SDHA*, and *SDHB* genes.

### Differential mRNA expression profiles between high- and low-grade chondrosarcoma

**Fig. 3:**
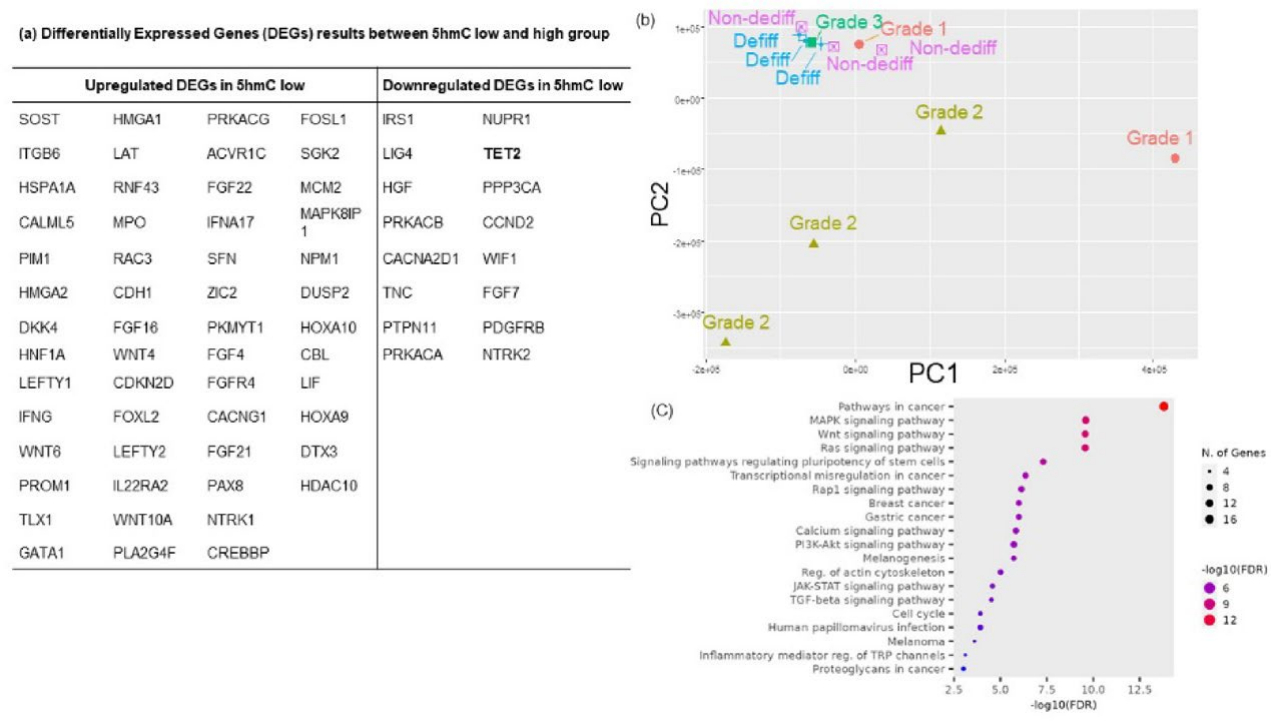
Differentially Expressed Genes (DEGs) results between 5hmC low and high group (**a**) and Principal Component Analysis of mRNA expression in chondrosarcoma (**b**). The non-dedifferentiated component in dedifferentiated chondrosarcoma (Non-dediff) has a similar RNA expression pattern to the dedifferentiated component (Dediff). Kyoto Encyclopedia of Genes and Genomes (KEGG) pathway analyses of the upregulated genes in 5hmC low compared to 5hmC high (**c**). These upregulated gene groups contain genes annotated with the mitogen-activated protein kinase and phosphatidylinositol-3 kinase pathways in KEGG datasets^15^.

The DV200 values for fresh-frozen specimens were 61%–75% (mean: 67.5%) whereas those for FFPE specimens were 29%–50% (mean: 37.4%). Notably, all FFPE samples had DV200 values above the recommended threshold of 30%, ensuring reliable data quality^14^. A comprehensive mRNA expression analysis was performed using nCounter®, and Principal Component Analysis (PCA) was performed (Fig. 3b). The contribution rate was 46% for the first principal component and 41.9% for the second principal component, resulting in a cumulative contribution of 87.9% for the first two principal components. Grade 3 CS and dedifferentiated CS exhibited similar expression patterns. Grade 1 and 2 cases, except for one grade 1 case, also showed similar expression patterns. The high-grade group, comprising grade 3 and dedifferentiated types, and the low-grade group, comprising grades 1 and 2, were mainly separated by the second principal component. The non-dedifferentiated component of dedifferentiated CS exhibited a similar expression pattern to the dedifferentiated component.

In the 5hmC low group, differentially expressed genes (54 upregulated genes and 16 downregulated genes) were identified (Fig. 3a). Among DNA methylation-related genes, the *TET2* gene was downregulated in the 5hmC low group. Gene Ontology analysis was performed to predict the functions of the upregulated genes in the 5hmC low group. The main biological process terms were “protein phosphorylation” (Supplementary Fig. S4). The results of KEGG pathway analysis are summarized in Fig. 3c.

### Reduced 5hmC levels are correlated with TET2 downregulation and activation of MAPK and PI3K/Akt/mTOR pathways

**Fig. 4:**
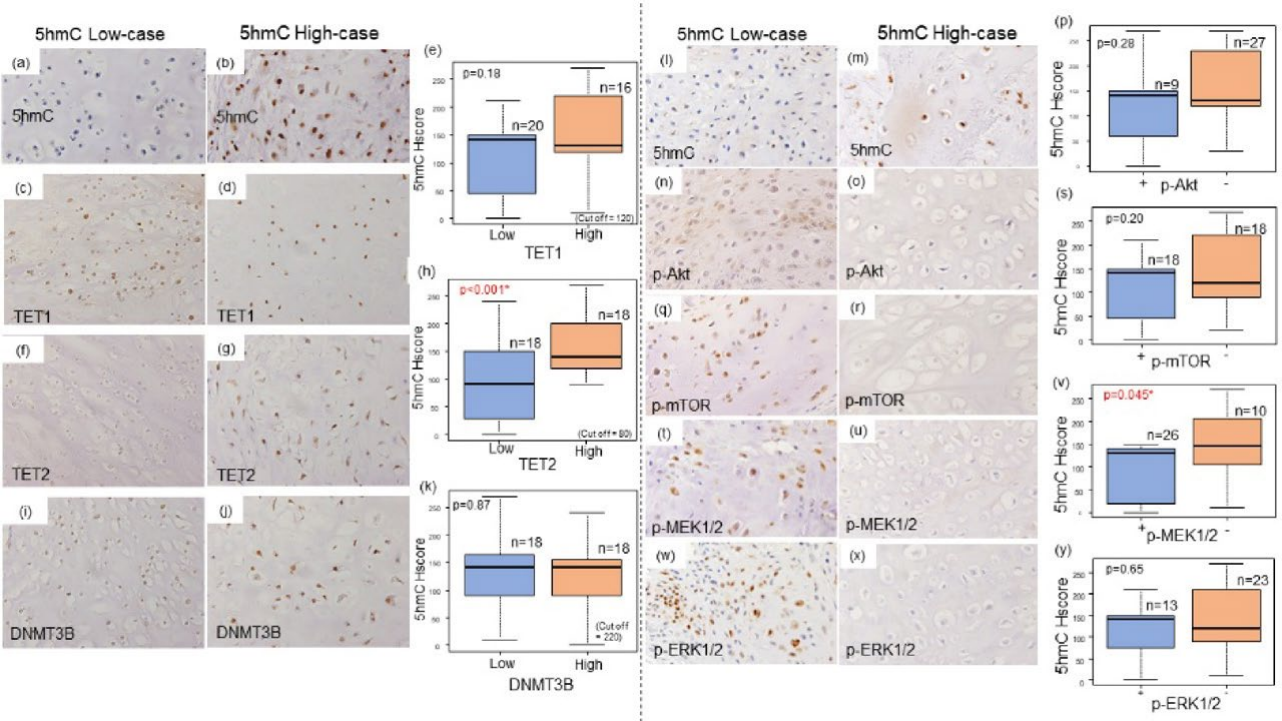
Relationship between TET1, TET2, and DNMT3B protein expression in the 5hmC low (**a**) and high (**b**) groups and immunohistochemical validation of p-Akt, p-mTOR, p-MEK1/2, and p-ERK1/2 immunostaining in chondrosarcoma between 5hmC high (**l**) and low (**m**) groups. In the low TET2 expression group, 5hmC levels were decreased (**f**, **g**, **h**) or tended to decrease with no statistically significant difference detected (**c**, **d**, **e**). No correlation was observed between DNMT3B (**i**, **j**, **k**) and 5hmC expression levels. Phosphorylated Akt (p-Akt) was confirmed positive in eight cases (**n**, **o**, **p**), p-mTOR in 17 cases (**q**, **r**, **s**), p-MEK in 26 cases (**t**, **u**, **v**), and p-ERK in 12 cases (**w**, **x**, **y**) out of 35 cases. The immunoexpression of p-MEK1/2 was significantly higher in the 5hmC low group than in the 5hmC high group (**v**). In the p-Akt, p-mTOR, and p-ERK1/2-positive group, the 5hmC expression levels tended to decrease (**p**, **s**, **y**).

IHC staining was performed to assess the protein expression of 5hmC, TET1, TET2, TET3, SDHB, DNMT3B, p-MEK1/2, p-ERK1/2, p-Akt, and p-mTOR in FFPE samples (Fig. 4, Supplementary S1). Based on KEGG enrichment analysis showing upregulation of the MAPK and PI3K/Akt/mTOR pathways in the low 5hmC group, we selected representative phospho-protein markers (p-Akt, p-MEK1/2, p-ERK1/2, and p-mTOR) for immunohistochemical validation. 5hmC, TET1, TET2, and DNMT3B were primarily expressed in the nuclei of tumor cells, while SDHB exhibited granular cytoplasmic staining. The median H-scores were 140 (IQR: 90–160) for 5hmC, 100 (IQR: 55–160) for TET1, 80 (IQR: 40–145) for TET2, and 210 (IQR: 190–270) for DNMT3B. Cutoff values for H-scores were set based on the median values as follows: 5hmC at 120, TET1 at 100, TET2 at 80, and DNMT3B at 220. SDHB showed positive staining in all cases, while TET3 was consistently negative. There was no significant association between 5hmC modification and histological grade (*p* = 0.608, Supplementary Fig. S5). 5hmC expression was significantly higher in the high TET2 expression group than in the low TET2 expression group. (*p* = 0.000529; Fig. 4). No significant association was observed between TET1, DNMT3B, and 5hmC expressions.

Staining for p-mTOR, p-MEK1/2, and ERK1/2 was observed in the nuclei, whereas staining for p-Akt was observed in both the nuclei and cytoplasm. p-Akt was positive in eight cases (25%), p-mTOR in 17 cases (49%), p-MEK1/2 in 26 cases (74%), and p-ERK in 12 cases (34%). In the high expression groups of p-MEK1/2, p-ERK1/2, p-Akt, and p-mTOR, the 5hmC expression tended to decrease, with a statistically significant difference observed in MEK1/2 (*p* = 0.045; Fig. 4).

### Lower 5hmC H-score reflects reduced 5hmC levels as confirmed by ELISA

The 5hmC content (%) was 0.00–0.415, with a median of 0.046. In three cases, 5hmC was undetectable. A strong positive correlation was observed between the 5hmC H-score and 5hmC content (%) (r = 0.813, Fig. S6).

### Low 5hmC levels and dedifferentiated chondrosarcoma predict poor survival

**Fig. 5:**
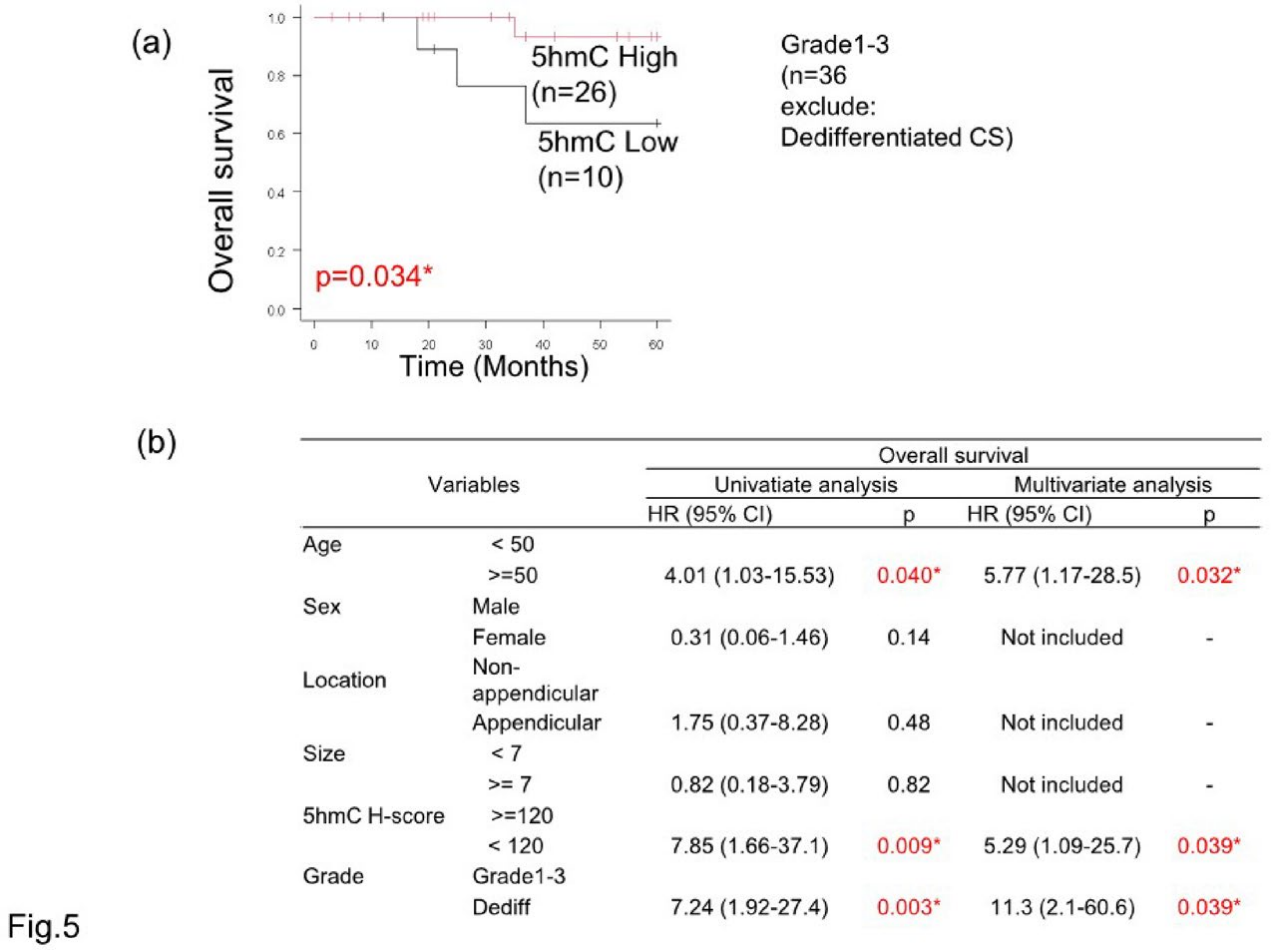
Kaplan–Meier plots of chondrosarcoma in the 5hmC high and low groups. Overall survival in chondrosarcomas grades 1–3 (**a**). The 5hmC low group had a poor prognosis. Results of univariate and multivariate analyses (**b**) showing age > 50, dedifferentiation type, and the 5hmC low group as poor prognostic factors.

In the univariate analysis, age of 50 or older (HR: 4.01, 95% CI: 1.03–15.53, *p* = 0.040), dedifferentiated type (HR: 7.24, 95% CI: 1.92–27.4, *p* = 0.003), and low 5hmC modification (HR: 7.85, 95% CI: 1.66–37.1, *p* = 0.009). In the multivariate analysis, age of 50 or older (HR: 5.77, 95% CI: 1.17–28.5, *p* = 0.032), dedifferentiated type (HR: 11.3, 95% CI: 2.1–60.6, *p* = 0.039), and low 5hmC modification (HR: 5.29, 95% CI: 1.09–25.7, *p* = 0.039) were identified as significant independent predictors of poor OS (Fig. 5). Survival curves using the Kaplan–Meier method are shown in Supplementary Figs. S2 and S3.

## Discussion

To our knowledge, this is the first study to demonstrate that reduced 5hmC modification represents an epigenetic alteration closely linked to CS malignancy. Although previous studies have reported 5hmC loss across various cancers, our study has been the first to establish its prognostic significance in CS. Notably, decreased 5hmC levels were correlated with poorer OS and PFS, highlighting 5hmC as a potential novel prognostic marker for CS.

Furthermore, our findings suggest that TET2 is involved in the regulation of 5hmC reduction in CS. The decreased 5hmC levels may lead to the activation of the MAPK and PI3K-Akt pathways, potentially contributing to the development of the malignant phenotype in CS. This interpretation is further supported by independent experimental studies demonstrating that TET2 deficiency promotes aberrant activation of PI3K/AKT and MAPK signaling^16,17^.

In this study, no correlation was observed between the WHO grade classification of CS and 5hmC levels, as variations in 5hmC levels were evident even within the same histological grade. The group with decreased 5hmC levels demonstrated a worse prognosis compared to the group without such a reduction. While reductions in 5hmC have been documented in various malignancies, including acute myeloid leukemia^18,19^, SDH-deficient gastrointestinal stromal tumor^20^, melanoma^21^, and other solid tumors^22,23^, this study is the first to establish an association between 5hmC modification and prognosis in CS. These findings suggest that, even in CS cases that appear histologically equivalent in malignancy, differences in methylation state accumulation may exist. As methylation accumulates, 5hmC modification decreases, leading to increased malignancy. Furthermore, a strong positive correlation was observed between the 5hmC H-score and quantitatively measured 5hmC levels, confirming that the H-score accurately reflects the actual 5hmC content. While Cleven et al. showed that IDH1/2 mutations did not correlate with 5hmC loss or prognosis in central chondrosarcomas, their study did not directly assess the prognostic significance of 5hmC levels. In contrast, our study found that reduced 5hmC expression was significantly associated with poor overall survival, suggesting that 5hmC may serve as a prognostic biomarker independent of IDH mutation status.

Therefore, the extent of 5hmC could potentially serve as an indicator of the malignancy stage, and IHC staining for 5hmC may be a valuable tool for assessing malignancy in CS.

To investigate factors contributing to the reduction in 5hmC levels, we performed IHC staining for TET1, TET2, TET3, SDHB, and DNMT3B. Our findings revealed that in the TET2 low-expression group, there was a concomitant reduction in 5hmC levels, indicating a potential role of TET2 in the regulation of demethylation in CS. Various mechanisms have been proposed to explain the reduction in 5hmC levels, including loss-of-function mutations in the *TET2* gene observed in hematologic malignancies^24,25^, as well as the inhibition of TET activity due to decreased α-ketoglutarate dehydrogenase and accumulation of 2-hydroxyglutarate resulting from *IDH1/2* mutations^26,27^. Despite conducting NGS analysis to identify gene mutations associated with the reduction in 5hmC levels, we did not find significant mutations in the *IDH* or *TET* genes that could account for this phenomenon. After investigating the effects of IDH1/2 mutations on DNA methylation in CS, Cleven et al. concluded that IDH mutations did not directly contribute to 5hmC loss^9^. However, even in the absence of gene mutations, the TET2 low-expression group still exhibited reduced 5hmC levels, suggesting the involvement of alternative mechanisms such as transcriptional inactivation or tumor hypoxia^28^ in downregulating *TET* gene expression. This is supported by previous observations of reduced TET2 expression in gliomas without *IDH* or *TET* gene mutations, with promoter methylation of the *TET2* gene proposed as an alternative mechanism for TET2 dysfunction^29^.

In mRNA expression analysis, PCA revealed that dedifferentiated CS and high-grade components like grade 3 exhibited similar expression patterns. Interestingly, the non-dedifferentiated components of dedifferentiated CSs, despite being histologically equivalent to grade 2, displayed an mRNA expression pattern more closely resembling the dedifferentiated components rather than the conventional CS grade 2. This raises the possibility that non-dedifferentiated components of dedifferentiated CS may harbor early molecular alterations distinct from conventional grade 2 CS, though further functional studies are needed to support this hypothesis. Previous molecular studies have demonstrated that both dedifferentiated and non-dedifferentiated tumor components share common genetic mutations, implying a single progenitor origin^30,31^. The PCA results support this finding. Accordingly, among cases with histologically grade 2 malignancy, there are those that can dedifferentiate and those that cannot. Therefore, some CS cases may not be appropriately classified per the WHO classification histological grade.

KEGG pathway analysis revealed that the 5hmC low group exhibited higher expression levels of genes associated with the MAPK and PI3K/Akt/mTOR pathways than the high group. Additionally, IHC analysis revealed higher expression levels of p-MEK1/2, p-ERK1/2, p-Akt, and p-mTOR in the 5hmC low group than in the high group. The MAPK pathway is well-known for its role in cancer development, promoting cell proliferation, resistance to apoptosis^32,33^, and metastasis in CS^34,35^. Furthermore, mTOR, a protein kinase in the PI3K/Akt/mTOR pathway, is a central regulator of metabolism and growth and is constitutively activated in various malignancies^36,37^. In CS, activation of the MAPK and PI3K/Akt/mTOR pathways is thought to enhance malignancy by promoting cell proliferation and angiogenesis.

TET2 is known for its role in activating the MAPK and PI3K/Akt/mTOR pathways. TET2 has been reported to regulate the ERK pathway, as seen in *TET2* knockout mice, where reduced 5hmC levels lead to overactivation of the ERK pathway due to reduced expression of the regulator Hspa1b, resulting in increased p-ERK levels^38^. In hepatocellular carcinoma, reduced TET2 levels are associated with decreased 5hmC levels and inhibition of histone acetyltransferases, resulting in excessive activation of the Akt signaling pathway^39^. These findings suggest that reduced TET2 expression in CS may trigger the activation of the MAPK and PI3K/Akt/mTOR pathways by reducing 5hmC levels, potentially affecting the expression of key regulators in these pathways.

Additionally, other studies have verified S6 phosphorylation, a surrogate marker for PI3K/mTOR activation, in 73 out of 106 (69%) conventional CSs and 11 out of 25 (44%) dedifferentiated CSs^40^. Based on these findings, it is conceivable that certain CSs with reduced 5hmC levels, resulting in the activation of the MAPK and PI3K/Akt/mTOR pathways, could potentially benefit from inhibitors.

This study has some limitations. First, given the low cellularity and abundant extracellular matrix in CS tissues, quantitative protein analysis via Western blot was challenging, leading to our reliance on IHC. Although IHC effectively determined protein localization, its quantitative precision is limited. However, we validated our findings using an ELISA-based approach, supporting the robustness of our results. Second, although our study demonstrated a clear association between 5hmC levels and CS progression, functional validation through knockdown or overexpression experiments was not performed. Although functional validation in cell-based models would be valuable, available chondrosarcoma cell lines are not suitable for grade-specific or dedifferentiated subtype analyses. Moreover, although previous studies have reported that 5hmC levels in chondrosarcoma are not associated with IDH1/2 mutations^9^, this could not be confirmed in the present study because only a subset of cases underwent sequencing and stratified analyses were not feasible. Finally, although reduced 5hmC expression was associated with poor prognosis, the absence of external validation, reproducibility testing, and comparison with established prognostic models indicates that these findings are preliminary. While small and unbalanced sample sizes reduce statistical power, this study still provides valuable insights into potential molecular alterations in chondrosarcoma. Future validation in larger cohorts will be essential to confirm and extend these findings. In conclusion, the reduction in 5hmC levels in CS plays a crucial role in promoting malignancy and can serve as a prognostic indicator for unfavorable outcomes. The observed association between reduced TET2 expression and lower 5hmC levels suggests a potential epigenetic mechanism contributing to activation of the MAPK and PI3K/Akt/mTOR pathways. Although the current data do not establish causality, these findings raise the possibility that TET2-related epigenetic changes may influence oncogenic signaling and tumor progression in chondrosarcoma. These associations should be interpreted with caution, and further mechanistic studies are needed to validate the functional relevance of the observed epigenetic and signaling alterations.

These findings are crucial for identifying potential treatment strategies for CS in the future.

## Supplementary Information

Below is the link to the electronic supplementary material.

Supplementary Material 1

## Data Availability

The datasets generated during and/or analyzed during the current study are available from Hiroshi Furukawa on reasonable request. The datasets generated and/or analyzed in this study are available in the NCBI Sequence Read Archive (SRA) repository under the BioProject accession number PRJNA1241222: https://www.ncbi.nlm.nih.gov/bioproject/1241222.
