# Supplementary material for: Reduced 5-hydroxymethylcytosine due to TET2 downregulation is associated with chondrosarcoma progression
Source: Sci Rep. 2025 Nov 25;15:41854. doi: 10.1038/s41598-025-25820-9 (PMC12647777; doi:10.1038/s41598-025-25820-9)
Supplement: Supplementary file 1 — Supplementary Material 1 [file 41598_2025_25820_MOESM1_ESM.pdf]

Supplementary materials

Summary of supplementary files

Table S1	Primary antibodies used for immunohistochemical staining.
Figure S1	Immunohistochemical images of 5hmC, TET1, DNMT3B and SDHB.
Figure S2,3	Kaplan–Meier curve for overall survival (OS) and progression free survival (PFS).
Figure S4	Gene Ontology (GO) analysis of the upregulated differentially expressed genes (DEGs) in the 5hmC low group than in the 5hmC high group.
Figure S5	Relation between 5hmC level (H-score) and histological grade.
Figure S6	Relationship between the 5hmC H-score and the total amount of 5hmC in DNA.

Table S1. Primary antibodies used for immunohistochemical staining.

Antibody	Company	Catalog number	Dilution
5hmC	Active Motif	39770	1:2000
TET1	abcam	ab191698	1:200
TET2	GeneTex	GTX124205	1:400
TET3	GeneTex	GTX121453	1:200
SDHB	SIGMA	HPA002868	1:100
DNMT3B	Santa Cruz biotechnology	sc-376043	1:200
p-Akt (Ser473)(736E11)	cell signaling	3787	1:100
Phospho-mTOR (ser2448)(49F9)	cell signaling	2976	1:50
Phospho-MEK1/2 (Ser221) (166F8)	cell signaling	2338	1:50
Phospho-p44/42 MAPK (Erk1/2)	cell signaling	9101	1:100

Figure S1.

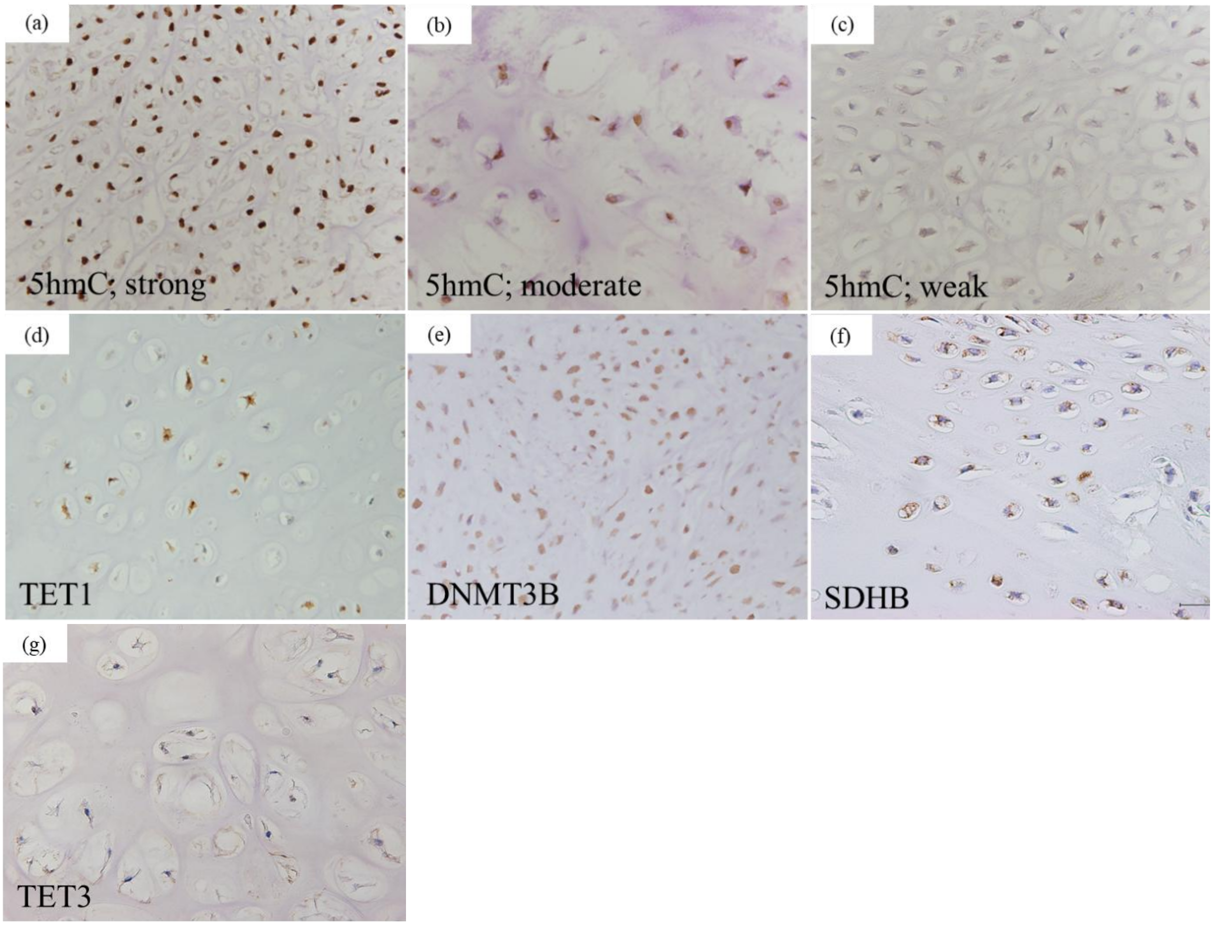

Figure S1. Immunohistochemical images of 5hmC, TET1, DNMT3B and SDHB.
Representative examples of 5hmC intensity score categories; strong (a) moderate (b) and weak (c).
TET1(d) and DNMT3B (e) also showed nuclear positive and SDHB was positive for cytoplasm (f).
SDHB expression was preserved in the all of the cases. TET3 was consistently negative (g).

Figure S2. Kaplan–Meier curves for overall survival (OS)

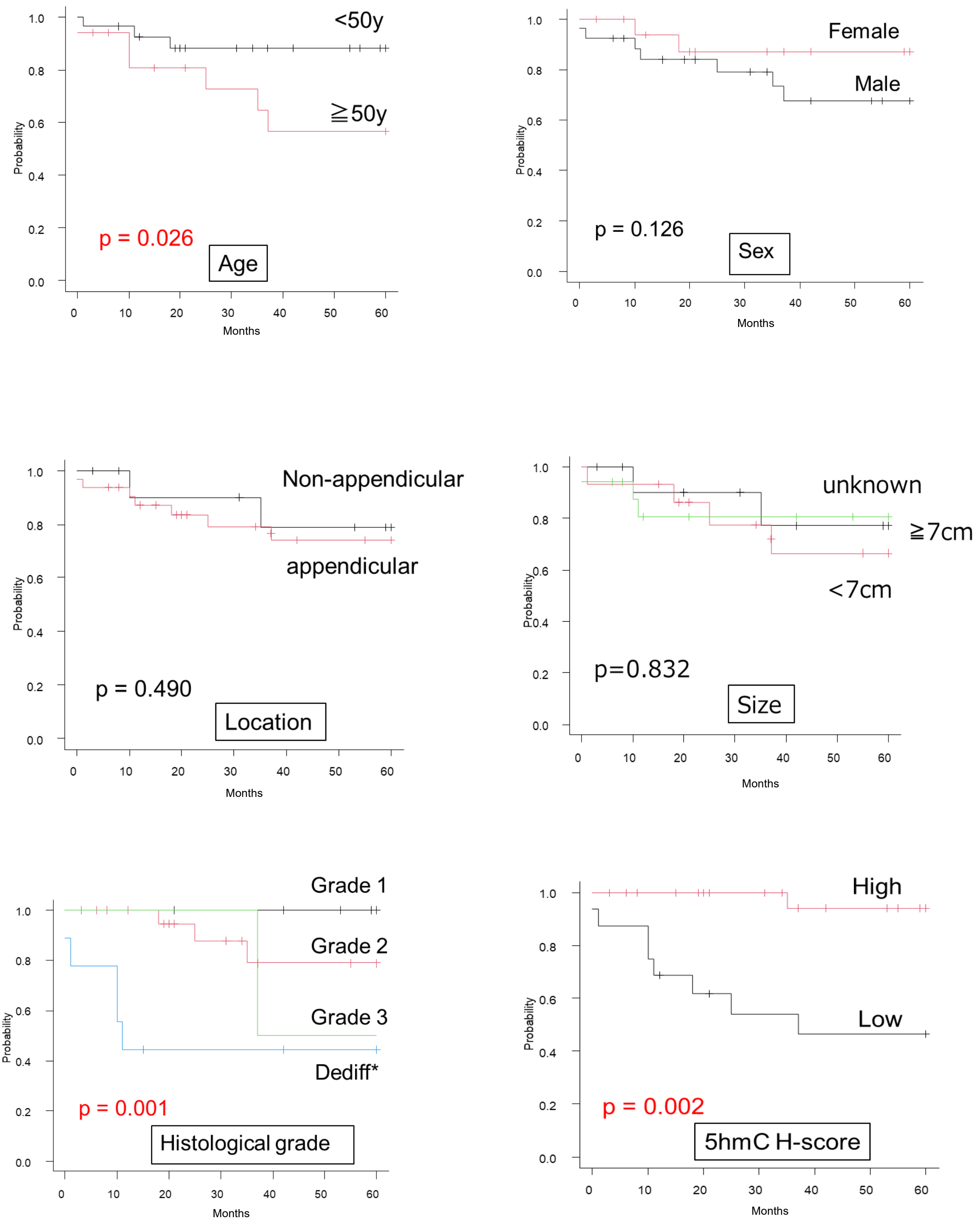

*Dedifferentiated chondrosarcoma

Figure S3. Kaplan–Meier curves for progression free survival (PFS)

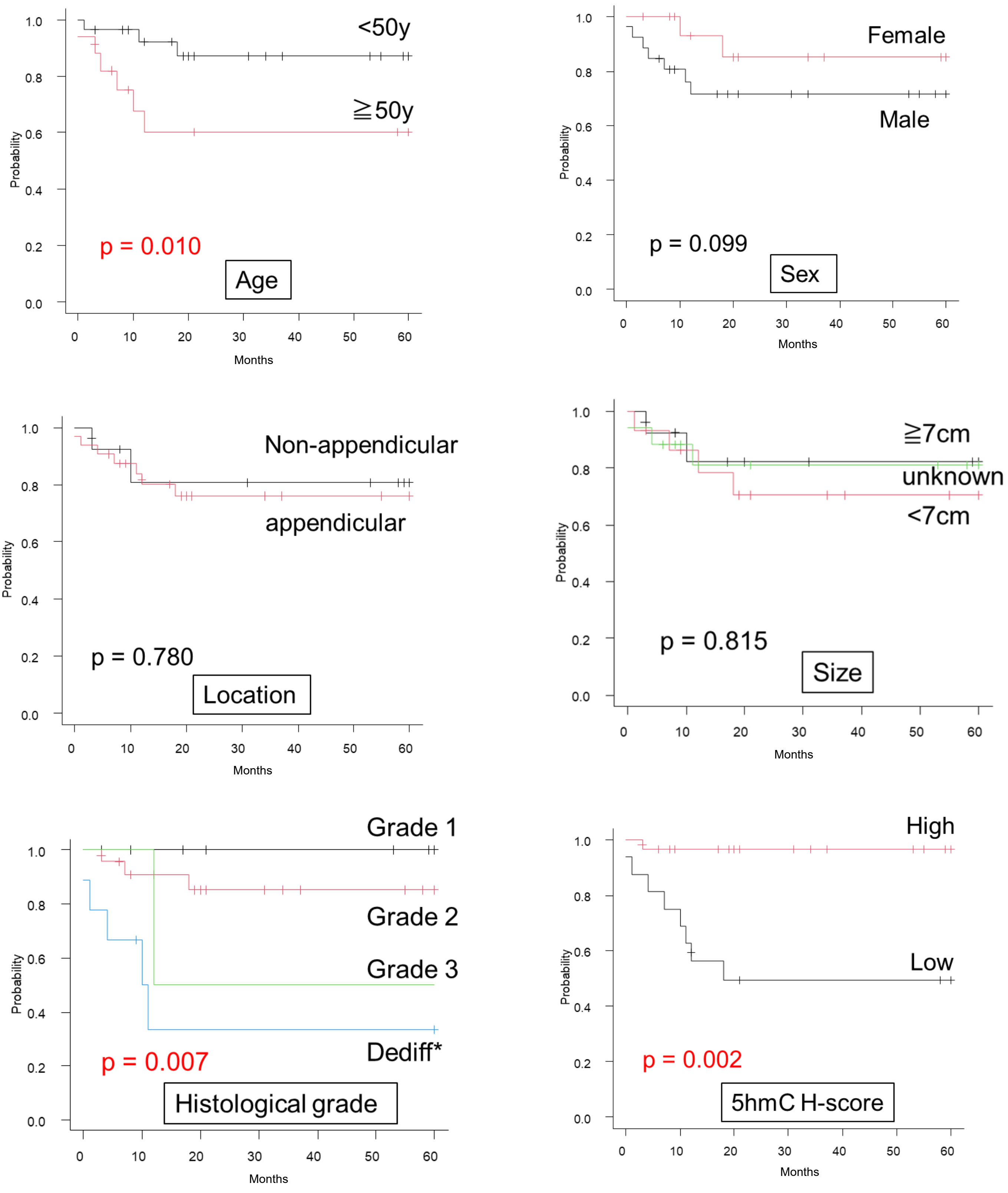

*Dedifferentiated chondrosarcoma

Figure S4.

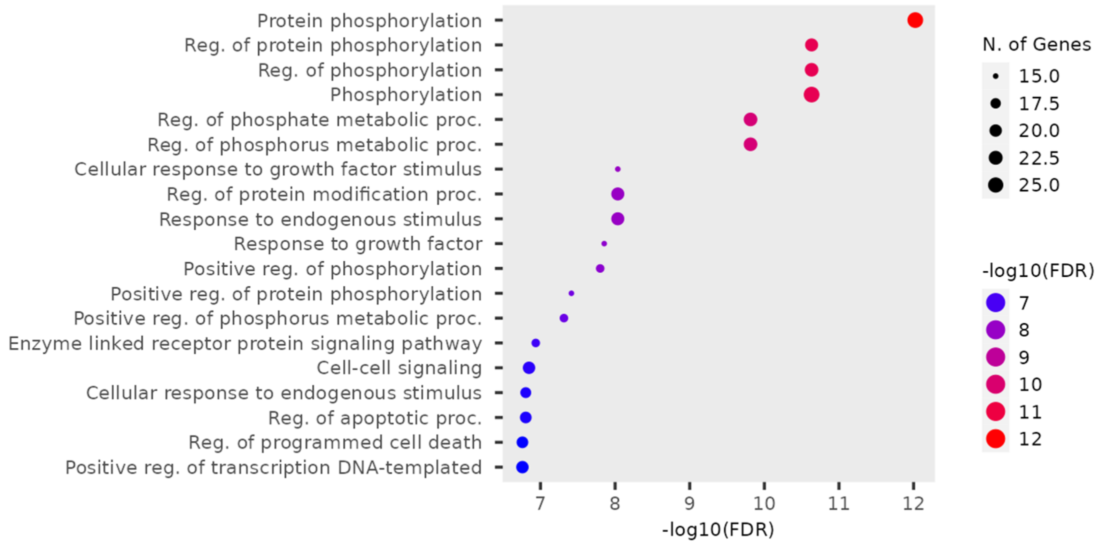

Figure S4. Gene Ontology (GO) analyses of the upregulated differentially expressed genes (DEGs) in the 5hmC low group than in the 5hmC high group.
The protein phosphorylation–related genes were upregulated in GO term of biological process.

Figure S5.

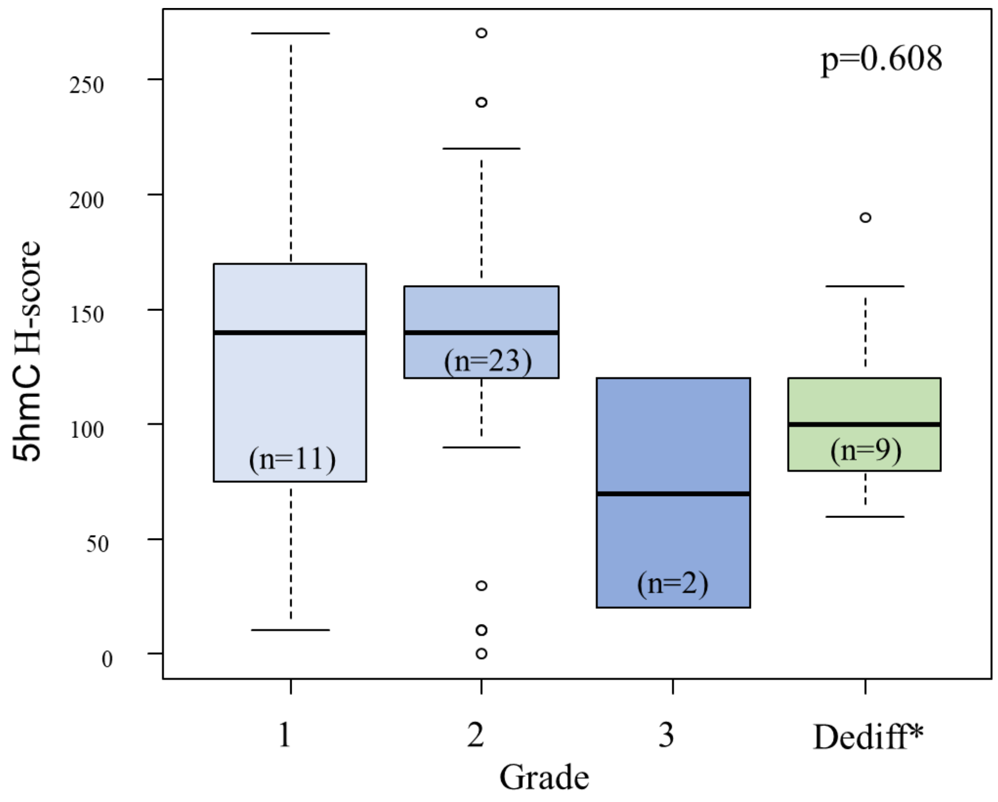

Figure S5. Relation between 5hmC level (H-score) and histological grade.
No correlation between 5hmC H-score and histological grade.
* Dedifferentiated component of dedifferentiated chondrosarcoma.

Figure S6.

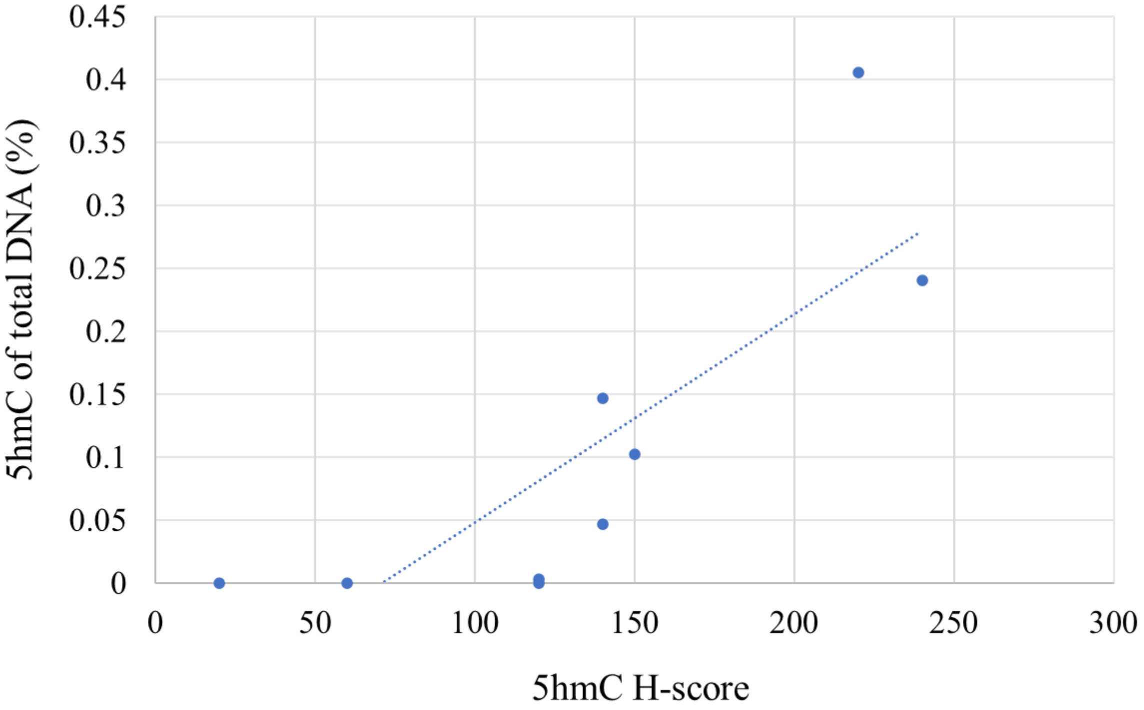

Figure S6. Relationship between the 5hmC H-score and the total amount of 5hmC in DNA. The correlation coefficient (r) was 0.81, indicating a strong positive correlation.
